# Neural oscillatory activity serving sensorimotor control is predicted by superoxide-sensitive mitochondrial redox environments

**DOI:** 10.1073/pnas.2104569118

**Published:** 2021-10-22

**Authors:** Rachel K. Spooner, Brittany K. Taylor, Iman M. Ahmad, Kelsey N. Dyball, Katy Emanuel, Howard S. Fox, Kelly L. Stauch, Matthew C. Zimmerman, Tony W. Wilson

**Affiliations:** ^a^Institute for Human Neuroscience, Boys Town National Research Hospital, Omaha, NE 68010;; ^b^College of Medicine, University of Nebraska Medical Center, Omaha, NE 68198;; ^c^College of Allied Health Professions, University of Nebraska Medical Center, Omaha, NE 68198;; ^d^Department of Neurological Sciences, University of Nebraska Medical Center, Omaha, NE 68198;; ^e^Department of Cellular and Integrative Physiology, University of Nebraska Medical Center, Omaha, NE 68198

**Keywords:** magnetoencephalography, Seahorse Analyzer, EPR spectroscopy, mitochondrial respiration, superoxide

## Abstract

Motor control requires a coordinated ensemble of spatiotemporally precise neural oscillations across a distributed motor network, particularly in the beta range (15 to 30 Hz) to successfully plan and execute volitional actions. While substantial evidence implicates beta activity as critical to motor control, the molecular processes supporting these microcircuits and their inherent oscillatory dynamics remain poorly understood. Among these processes are mitochondrial integrity and the associated redox environments, although their direct impact on human neurophysiological function is unknown. Herein, 40 healthy adults completed a motor sequence paradigm during magnetoencephalography (MEG). MEG data were imaged in the time–frequency domain using a beamformer to evaluate beta oscillatory profiles during distinct phases of motor control (i.e., planning and execution) and subsequent behavior. To comprehensively quantify features of the mitochondrial redox environment, we used state-of-the-art systems biology approaches including Seahorse Analyzer to assess mitochondrial respiration and electron paramagnetic resonance spectroscopy to measure superoxide levels in whole blood as well as antioxidant activity assays. Using structural equation modeling, we tested the relationship between mitochondrial function and sensorimotor brain-behavior dynamics through alterations in the redox environment (e.g., generation of superoxide and alteration in antioxidant defenses). Our results indicated that superoxide-sensitive but not hydrogen peroxide–sensitive features of the redox environment had direct and mediating effects on the bioenergetic–neural pathways serving motor performance in healthy adults. Importantly, our results suggest that alterations in the redox environment may directly impact behavior above and beyond mitochondrial respiratory capacities alone and further may be effective targets for age- and disease-related declines in cognitive–motor function.

## Significance

Mitochondrial integrity and associated redox profiles have long been revered as key contributors to a host of age- and disease-related pathologies, which eventually lead to neuronal and behavioral dysfunction in the sensorimotor and other systems. However, the precise role of the mitochondrial redox environment in human sensorimotor brain systems and motor behavior remains poorly understood. Herein, we provide evidence for a strong predictive capacity of superoxide and its scavenger, superoxide dismutase, on the neural oscillatory dynamics serving motor planning and execution above and beyond the effects of mitochondrial respiratory capacities alone. Importantly, these data provide insight regarding the impact of the redox environment on the population-level neural oscillations that serve motor function in healthy humans.

Motor control requires desired movement kinematics to be transformed into discrete plans to successfully execute volitional actions. In the brain, the planning and execution of volitional movement is implemented through multispectral, population-level neural oscillatory activity, particularly in the beta band (e.g., 15 to 30 Hz). Specifically, robust beta responses typically emerge several hundred milliseconds before movement onset, extend through movement execution, and are believed to reflect the active engagement of the motor network during planning and execution periods in the bilateral primary motor cortices (M1) and secondary motor regions (1–4). Importantly, while prior work suggests that this pattern of activity is not particularly susceptible to changes in the movement kinematics themselves (e.g., speed, force applied, and muscle groups engaged) (5), beta power has been shown to be especially pertinent to higher-order planning and movement selection factors such as response certainty (1–3, 6, 7) and movement complexity (8), making its role in behavioral modulation to achieve desired outcomes profoundly important. Further, these well-established sensorimotor brain-behavior dynamics in humans appear to be extremely sensitive to participant characteristics such as chronological age and neurodegeneration (9–11), although the precise mechanisms serving individual alterations in sensorimotor brain-behavior trajectories remains unclear.

One proposed contributor to the neural and behavioral variability observed in health and disease is the bioenergetic capacity of the mitochondria and associated redox environment (12–14). The importance of mitochondria in an organ as energetically expensive as the brain is highlighted by its increased dysregulation in a host of neurodegenerative diseases, including Alzheimer’s disease (15), Parkinson’s disease (16), and even healthy aging (13), often leading to cognitive and behavioral abnormalities observed most commonly in animal models. Essentially, neuronal function (e.g., synaptic vesicle release and neurotransmitter release) is known to be regulated by mitochondria through various mechanisms, including changes in mitochondrial motility, morphology, ATP production, and oxygen consumption (14, 17, 18), albeit its role in human neurophysiology and behavior is less well understood. Furthermore, as a result of these changes to mitochondrial structure and function, the generation of reactive oxygen species (ROS; e.g., superoxide and hydrogen peroxide [H_2_O_2_]), concomitant with alterations in their antioxidant scavengers (e.g., superoxide dismutase [SOD], catalase, and glutathione), may also contribute to functional aberrations in brain and behavior (19, 20). For example, in aging mice, those with damage to important antioxidant defenses (i.e., SOD knock out models) exhibit significant deficits in sensory and motor neural functions (e.g., tail flick latency) (21), implicating a role for superoxide-sensitive mechanisms in the peripheral and central nervous system. Importantly, mitochondrial function and discrete alterations to the redox environment have also been shown to differentially modulate inhibitory interneuronal pools in animal models of psychiatric and neurodegenerative disorders (22–25). This is of particular interest to the current study, as substantial evidence implicates inhibitory interneuronal drive on local pyramidal cells as critical to population-level oscillatory responses, including the beta oscillations pertinent to human sensorimotor control (26–33). Taken together, these data suggest that evaluation of the mitochondrial redox environment may provide insight into changes in the oscillatory brain dynamics pertinent to healthy human behavior and sensorimotor processing.

The key goals of the current study were to comprehensively quantify parameters of mitochondrial function and the redox environment and to assess their predictive capacity on the neuronal functions serving sensorimotor performance. To this end, 40 healthy adults completed a movement sequence paradigm during magnetoencephalography (MEG) to characterize beta oscillatory activity during distinct phases of motor control (i.e., planning and execution). To facilitate comparison with the majority of past literature, we aimed to evaluate the impact of mitochondrial redox biology on trial-averaged neural beta oscillations within the contralateral M1 during the planning and execution phases of movement. However, several recent studies have suggested that these temporally sustained beta oscillations in M1 cortex may actually be comprised of numerous transient “bursts” of beta activity (34). The notion that such beta oscillations should be conceptualized in a bursting framework has been studied across sensory modalities (e.g., somatosensory and motor), species (e.g., animals and humans), and clinical populations (e.g., Parkinson’s disease). Further, premovement (i.e., baseline) burst event parameters (e.g., peak event power and event count/rate) may be a consistent modulator of behavioral performance (34–39). Thus, to complement our analysis of the widely studied, temporally sustained beta oscillations, we conducted supplementary analyses within the beta-burst framework. Finally, we used state-of-the-art systems biology approaches to directly quantify real-time mitochondrial respiration using Seahorse Analyzer of mitochondrial stress tests, total intracellular superoxide levels using electron paramagnetic resonance (EPR) spectroscopy, and numerous antioxidant assays sensitive to superoxide and H_2_O_2_ redox pathways in the periphery. Of note, recent evidence suggests that bioenergetic features of the redox environment (e.g., mitochondrial respiration) measured in the periphery correspond closely to those measured from isolated mitochondria in the central nervous system (40), making the evaluation of blood-based markers an attractive avenue for investigation in humans. Using structural equation modeling, we tested the hypothesis that changes in the bioenergetic capacity of the mitochondria (i.e., spare respiratory capacity [SRC]) would be differentially predictive of redox environments (i.e., ROS concentration and antioxidant defenses) sensitive to superoxide and H_2_O_2_ pathways, separately. In addition, we hypothesized that the generation of ROS in concert with increased antioxidant defenses would differentially predict neural oscillatory activity during the planning and execution phases of movement, with significant impacts on subsequent behavioral task performance. Importantly, our results suggest that superoxide-sensitive but not H_2_O_2_-sensitive redox mechanisms robustly predict brain-behavior dynamics in the contralateral primary motor cortex (M1) during motor control in healthy adults.

## Results

The planning and execution of volitional actions is known to be associated with decreases in beta oscillatory activity in the sensorimotor network. However, the molecular processes that may modulate individual performance and the associated brain-behavior dynamics are unclear, although the mitochondrial redox environment likely contributes. Thus, we investigate this linkage using state-of-the-art MEG imaging and systems biology approaches.

### Sequential Movement Performance.

**Fig. 1.**
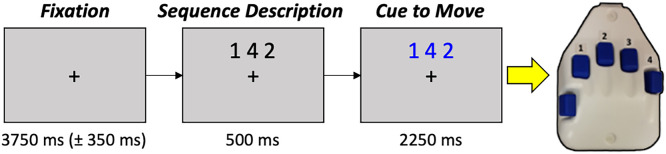
Motor sequence paradigm. Participants were instructed to fixate on a centrally located crosshair for 3,750 ms (±350 ms), followed by a sequence description (500-ms duration) consisting of three numbers corresponding to fingers on the right hand (reference the right-handed button pad to the far right). The cue to move was indicated by the numbers changing color to blue. Participants were instructed to tap the sequence in order as quickly and accurately as possible following the color change.

Following removal of trials with anomalous performance (e.g., reaction times >1,250 ms; movement durations >3,000 ms), an average of 123 trials per participant remained for subsequent statistical modeling. Participants performed generally well on the task (Fig. 1), with an average accuracy of 93.93% (SD = 4.69%). Average reaction time (i.e., response time to complete the first button press) was 479.17 ms (SD = 137.91 ms), while the average movement duration (i.e., time to complete the entire motor sequence) was 831.44 ms (SD = 234.47 ms), which aligns well with prior studies of healthy adults (8).

### MEG Sensor- and Source-Level Analyses.

**Fig. 2.**
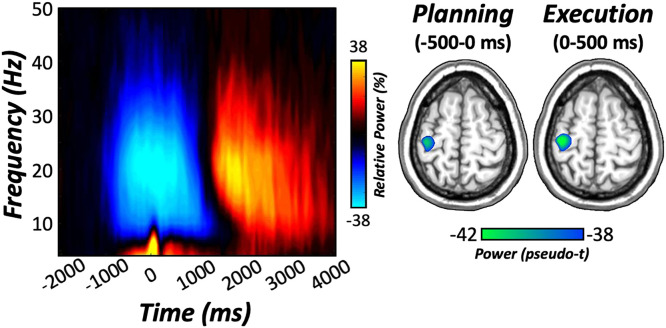
Movement-related oscillations at the sensor and source level. (*Left*) Time–frequency spectrograms locked to the onset of the first movement in the sequence (0 ms) for a sensor near the left sensorimotor cortex. The *x*-axis denotes time (in milliseconds), and the *y*-axis denotes frequency (in Hertz). Power expressed as percent change from baseline (−2,050 to −1,550 ms) is shown in the color scale bar to the right of the graphic. (*Right*) Significant peri-movement decreases in beta oscillatory activity (16 to 26 Hz) were observed during motor planning (−500 to 0 ms) and execution (0 to 500 ms) phases in the left M1 contralateral to movement. *n* = 40.

Time–frequency analyses of movement-locked MEG data (i.e., locked to the first movement in the sequence) indicated significant multispectral peri- and postmovement oscillatory responses (Fig. 2). These responses were robust in gradiometers near the contralateral sensorimotor strip across all participants (*P* < 0.001, Fig. 2). Specifically, decreases in peri-movement beta activity (16 to 26 Hz) were observed prior to and following movement onset (i.e., −500 to 0 ms and 0 to 500 ms, respectively). In addition, we observed transient increases in theta activity near movement onset as well as weaker sustained decreases in the alpha range (10 to 14 Hz; Fig. 2). Movement-related gamma oscillations were not reliably detected (*SI Appendix*). Finally, we observed postmovement increases in beta activity following motor termination. However, given the known role of beta oscillations in motor planning and performance, we focused our primary analyses on these responses; results pertaining to the other oscillatory responses are included in *SI Appendix*.

To identify the neural origins of oscillations detected during the discrete phases of motor control, planning and execution windows were imaged separately using a beamformer. The resulting maps indicated that peri-movement decreases in beta power originated from the left M1 contralateral to movement (Fig. 2), with identical peak locations observed for motor planning (i.e., −500 to 0 ms) and execution (i.e., 0 to 500 ms) phases. Peak voxel values (i.e., pseudot) were then extracted from the left M1 per time bin, and these were used for subsequent statistical modeling. In addition, time series were extracted from the peak voxel and used to compute the beta event count and peak event power, which are the most-common metrics in the beta bursting framework (35, 39). These two bursting parameters were used in statistical models parallel to those computed for the more common beta oscillatory strength (see *Statistical Analysis* in the* Materials and Methods* section below). However, no significant relationships with the mitochondrial redox environment were detected for the bursting parameters, and thus, these models are reported in *SI Appendix*.

### Superoxide-Sensitive Redox Environments Predict Movement-Related Dynamics.

**Fig. 3.**
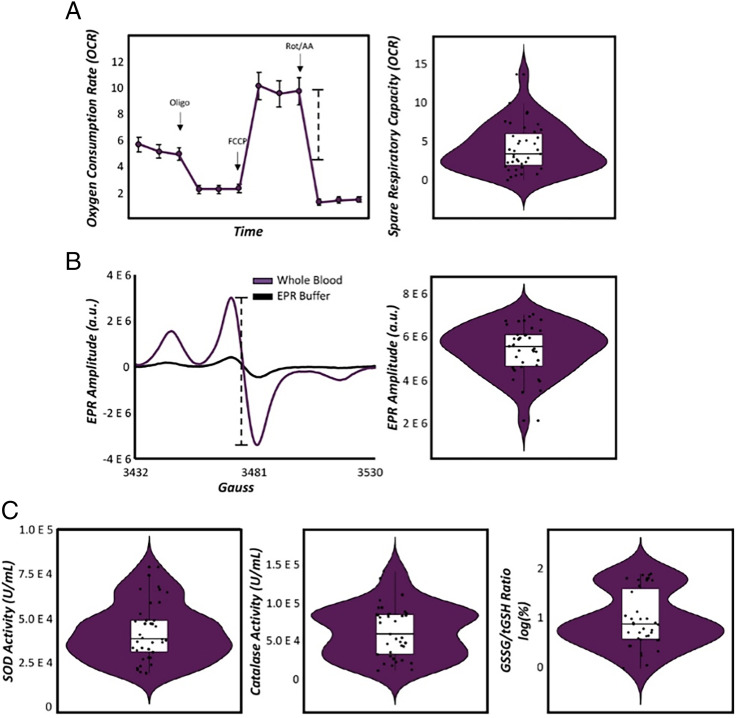
Mitochondrial redox environment in healthy adults. (*A*) OCRs were recorded using Seahorse XF96 Analyzer of PBMCs during serial injections of electron transport chain inhibitors (i.e., oligo, rot/AA) and a mitochondrial oxidative phosphorylation uncoupler (i.e., FCCP) to quantify mitochondrial respiration. The dashed line denotes the calculation of SRC (i.e., maximal respiration—basal respiration) and is indicative of the mitochondria’s ability to adapt to changing energy demands (shown in combined violin and box plot to the right). (*B*) EPR spectra of whole blood (shown in purple) incubated with a superoxide-sensitive spin probe compared to EPR buffer incubated with the same spin probe (shown in black) for a representative subject. The *x*-axis denotes magnetic field strength (in gauss) while the *y*-axis denotes EPR amplitude (in arbitrary units [a.u.]). Absolute peak-to-trough distance (denoted in the dashed line) is directly proportional to the total amount of cellular superoxide (shown in combined violin and box plot to the right). (*C*) Violin/box plots for commercially available antioxidant activity assays in red blood cells (RBCs) for SOD, catalase, and GSSG/tGSH ratios (i.e., the reducing capacity of glutathione). *n* = 40.

**Fig. 4.**
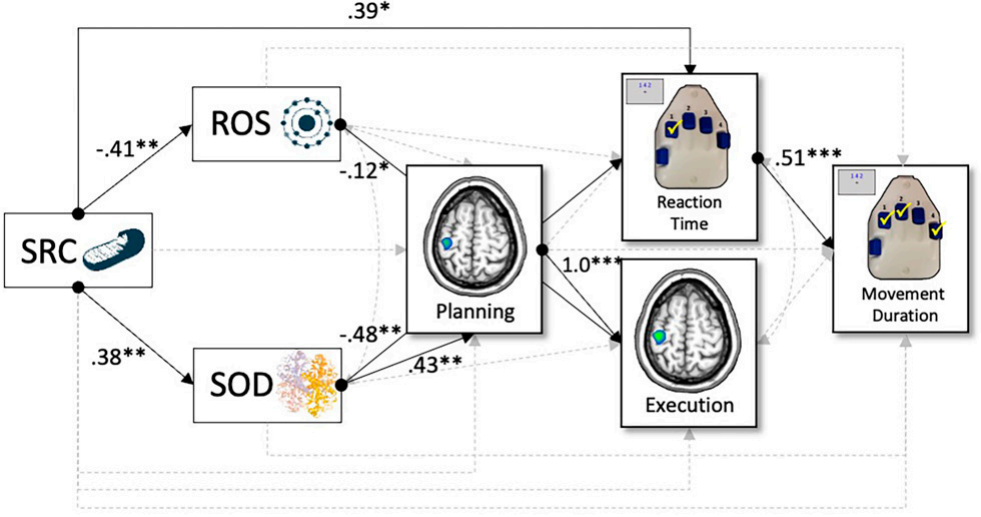
Superoxide-sensitive mitochondrial redox environments predict neural oscillations serving motor behavior. Results of the structural equation model probing the predictive capacity of superoxide-sensitive mitochondrial redox environments on movement-related oscillations and behavior. Single-headed arrows denote predictive paths, and double-headed arrows denote correlations. Statistically significant estimates (*P* < 0.05) are denoted with solid lines, and single-headed arrows with circles represent the beginning of a significant path, while nonsignificant paths are denoted with dashed gray lines. All listed parameters are standardized coefficients. *P < 0.05, **P < 0.01, and ***P < 0.001. *n* = 40.

We tested a multiple-mediation model (*Materials and Methods*) whereby measures of the mitochondrial redox environment sensitive to superoxide (i.e., SRC, superoxide, and SOD activity; Fig. 3) predicted brain-behavior relationships serving motor performance. Specifically, our possible brain-behavior relationships included paths whereby beta oscillatory activity during motor planning (−500 to 0 ms) predicted reaction time, as well as beta activity during motor execution (0 to 500 ms). Variables during planning and execution phases then predicted movement duration (i.e., time to complete the entire sequence). For a full model visualization, refer to Fig. 4.

As expected, reaction time was a robust predictor of movement duration, such that faster responses to the first number in the sequence were associated with faster sequence completion time (β = 0.51, *P* < 0.001). In addition, beta activity during the planning period strongly predicted activity during the execution phase in the left M1 contralateral to movement, such that greater decreases in beta oscillatory activity prior to movement were predictive of greater decreases during sequence execution (β = 1.0, *P* < 0.001). Interestingly, while beta activity prior to movement did not significantly predict reaction time nor movement duration (*ps* > 0.109), weaker beta responses during motor execution were marginally predictive of faster sequence completion times (i.e., movement duration; β = −1.09, *P* = 0.061).

In regard to superoxide-sensitive redox environments, we observed no direct effects of mitochondrial function on peri-movement oscillatory activity in the contralateral M1 nor movement duration (*ps* > 0.105), although decreases in SRC were predictive of faster reaction times (β = 0.39, *P* = 0.027; Fig. 4). In contrast, we observed robust direct effects of the redox environment on brain and behavioral function. Specifically, levels of cellular superoxide were robust predictors of beta activity during motor execution, such that increased levels of superoxide were predictive of weaker beta responses (β = −0.12, *P* = 0.012; Fig. 4), while increased activity levels of superoxide’s scavenger (i.e., SOD) were predictive of weaker planning-related beta responses in the left M1 (β = 0.43, *P* = 0.004) and faster reaction times on the motor sequence task (β = −0.48, *P* = 0.003).

Next, we examined all potential mediating effects of the redox environment on mitochondrial-related changes in motor cortical dynamics and behavioral performance. As described in *Materials and Methods*, statistical significance of these indirect effects were determined by using bias-corrected bootstrapped confidence intervals (41, 42); thus, exact *P* values are not available. In total, we observed five statistically significant indirect effects (Table 1, all *p*s < 0.05). First, there was a full mediation of mitochondrial SRC on movement duration through levels of SOD and reaction time (β_indirect_ = −0.09, b_indirect_ = −7.41, 95% CI: [−26.55, −0.95]), such that increases in SRC were associated with increases in SOD activity levels (β = 0.38, *P* = 0.013), which was associated with faster reaction times (β = −0.48, *P* = 0.006) and subsequently shorter movement durations to complete the entire motor sequence (β = 0.51, *P* = 0.001). Similarly, the relationship between mitochondrial function and reaction time was partially mediated by levels of SOD activity (β_indirect_ = −0.18, b_indirect_ = −8.62, 95% CI: [−23.51, −2.27]), such that increases in SRC were associated with increases in SOD activity levels and faster reaction times. In regard to the brain, planning- and execution-related beta oscillations were mediated by measures of the redox environment through disparate mechanisms, such that superoxide was a partial mediator of the relationship between SRC and neural activity during motor execution (β_indirect_ = 0.05, b_indirect_ = 0.51, 95% CI: [0.01, 1.56]), while SOD partially mediated changes in planning-related neural oscillations (β_indirect_ = 0.16, b_indirect_ = 1.67, 95% CI: [0.15, 4.71]). Interestingly, decreases in mitochondrial SRC were associated with increased levels of superoxide (β = −0.41, *P* = 0.005), which were associated with weaker execution-related decreases in beta activity in the contralateral M1 (β = −0.12, *P* = 0.007). In contrast, when accounting for increasing levels of SOD, we observed a multiple mediation of the relationship between mitochondrial SRC and execution-related beta oscillations through planning-related decreases in the contralateral M1 (β_indirect_ = 0.16, b_indirect_ = 1.67, 95% CI: [0.16, 4.84]). Essentially, greater mitochondrial energetic reserve was associated with increased activity levels of SOD (β = 0.38, *P* = 0.013), which significantly predicted weaker planning-related beta power (β = 0.43, *P* = 0.008) and subsequent beta decreases during movement execution in the contralateral M1 (β = 1.0, *P* < 0.001). Importantly, superoxide-sensitive features of the redox environment and mitochondrial function accounted for 34.9% of the variance in movement duration (*P* < 0.001).

**Table 1.** Results of the mediation analyses of mitochondrial function on brain behavior through superoxide-pertinent redox parameters

Mitochondrial SRC to movement duration			
Path	Total	Direct	Indirect
SRC→SOD→RT→MD	0.13	0.02	−0.09*
Mitochondrial SRC to reaction time			
SRC→SOD→RT	0.16*	0.39*	−0.18*
Mitochondrial SRC to motor execution			
SRC→EPR→Exec	−0.13*	−0.08*	0.05*
SRC→SOD→Plan→Exec	−0.13*	−0.08*	0.16*
Mitochondrial SRC to motor planning			
SRC→SOD→Plan	−0.09	−0.19*	0.16*

Indirect effects were assessed for statistical significance using the bias-corrected bootstrapped CIs. All reported parameters are standardized coefficients. *P < 0.05.

### H_2_O_2_-Sensitive Pathways Do Not Predict Motor Cortical Dynamics and Behavior.

**Fig. 5.**
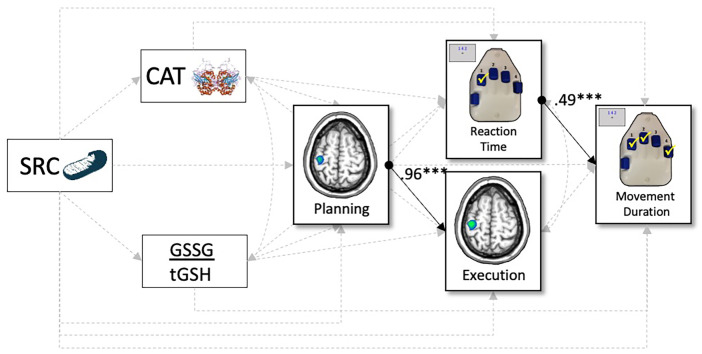
H_2_O_2_-sensitive mitochondrial redox environments do not predict movement-related brain-behavior relationships. Results of the structural equation model probing the predictive capacity of H_2_O_2_-sensitive mitochondrial redox environments on movement-related oscillations and behavior. Single-headed arrows denote predictive paths, and double-headed arrows denote correlations. Statistically significant estimates (*P* < 0.05) are denoted with solid lines, and single-headed arrows with circles represent the beginning of a significant path, while nonsignificant paths are denoted with dashed gray lines. All listed parameters are standardized coefficients. ***P < 0.001. *n* = 40.

Next, we evaluated the predictive capacity of H_2_O_2_-sensitive redox environments and mitochondrial function on brain-behavior relationships serving sensorimotor control using the same regression equation described above (see *Superoxide-Sensitive Redox Environments Predict Movement-Related Dynamics*). However, in this model, our measures of the redox environment were comprised of catalase activity and GSSG/tGSH ratios as continuous predictors of movement-related beta activity during motor planning and execution phases, as well as behavior on the task (see Fig. 5).

As with the first model, reaction time was a robust predictor of movement durations (β = 0.49, *P* < 0.001; Fig. 5), such that faster responses to the first number in the sequence led to shorter times to complete the full movement. In regard to the brain, movement-related beta oscillations during the planning period were robust modulators of execution-related decreases in beta activity (β = 0.96, *P* < 0.001; Fig. 5). In addition, weaker oscillatory activity during motor execution in the contralateral M1 was marginally predictive of shorter movement completion times (β = −0.99, *P* = 0.065). As per H_2_O_2_-sensitive mitochondrial redox environments, we observed no direct effects of mitochondrial SRC nor H_2_O_2_-sensitive antioxidants on brain or behavioral indices in our sample. In addition, we observed no mediating effects of the redox environment between mitochondrial function, neural, or behavioral metrics.

## Discussion

In the current study, we used advanced neurophysiology and systems biology approaches to evaluate the predictive capacity of mitochondrial function and the redox environment on motor cortical function and behavioral performance in a large sample of healthy adults. Specifically, we observed robust direct effects of superoxide-sensitive redox environments, such that increases in superoxide were significant predictors of execution-related beta oscillations in the contralateral M1, while increases in SOD were predictive of planning-related beta power. In addition, both superoxide and SOD were strong mediators of the relationship between mitochondrial energetic reserve and brain-behavior relationships serving sensorimotor function. Taken together, superoxide-sensitive features of the mitochondrial redox environment accounted for a substantial proportion of the variance (34.9%) in movement duration. Finally, our data suggest that unlike superoxide-related mechanisms, features involved in H_2_O_2_-pertinent redox pathways were not predictors of sensorimotor brain-behavior dynamics in healthy adults. Below, we discuss the implications of these findings for understanding the contribution of mitochondrial redox environments in human neurophysiology and sensorimotor control.

Our most important finding was likely the contribution of superoxide-sensitive features of the redox environment to the neural dynamics serving sensorimotor control in humans. Specifically, increases in superoxide were robust predictors of weaker beta oscillations in the M1 contralateral to movement during motor execution. In contrast, increases in total SOD (i.e., the antioxidant important for scavenging superoxide) were associated with weaker beta oscillations during the planning phase and faster reaction times on the task. Importantly, these results coincided with a well-established pattern of findings in the healthy motor system, such that weaker planning-related beta oscillations in M1 were predictive of weaker beta oscillations during motor execution and shorter movement durations on the task (i.e., time to complete the entire sequence) (9, 43). The idea that superoxide-sensitive redox pathways modulate motor cortical oscillations and subsequent performance in humans is not surprising, albeit prior work has solely interrogated these relationships in animal models. For example, deficiencies in the cytosolic isoform of SOD (i.e., SOD1) have long been revered as key contributors to age- and disease-related functional dependencies, with the most-common demonstration of this seen in mouse models of amyotrophic lateral sclerosis (ALS) (44). Essentially, gene mutations in SOD1 lead to sharp reductions in SOD activity levels, which in turn results in increased levels of ROS that give rise to the progressive atrophy and paralysis seen in motor neuron diseases (i.e., ALS) (44, 45). In addition, reductions in SOD activity levels have been linked to the healthy aging process in mice, such that increasing age is associated with reduced SOD activity, greater ROS production (e.g., lipid peroxidation, nitrated proteins, and superoxide generation), and impaired functionality (e.g., reduced exploration time and increased tail flick latency) (21, 46, 47). Importantly, our study describes the modulation of sensorimotor brain-behavior relationships by the redox environment in normative human physiology, and further, our results suggest that in healthy systems, increases in SOD and superoxide are associated with more optimal oscillatory response profiles and better motor performance. Furthermore, these relationships appeared to be specific to beta frequency oscillations during motor planning and execution and were not observed with other motor-related oscillations or bursts.

In agreement with the notion that superoxide-sensitive redox pathways govern more optimal brain-behavior dynamics, we observed robust mediations of the mitochondrial bioenergetic–neural pathway when accounting for increasing levels of superoxide and SOD. Contrary to our hypotheses, we observed no direct effects of mitochondrial SRC on movement-related cortical oscillations nor movement duration. This was somewhat surprising, as there is substantial evidence implicating mitochondrial functionality in modulating brain-behavior dynamics in animals (12, 13, 18, 20). For example, Hara et al. demonstrated a relationship between mitochondrial morphology and content with better working memory performance in aged monkeys, such that increased total number of straight mitochondria were associated with better accuracy on the task (18). Conversely, increased prevalence of donut and blob-shaped mitochondria in presynaptic boutons (i.e., a marker of oxidative stress) were associated with smaller active zones, fewer docked synaptic vesicle pools, and working memory impairment (18). In a different study, Khacho and colleagues observed discrete alterations to motor, learning, and memory function (i.e., reduced time on the rotarod, inability to improve over trials, and increased latency to platform in Morris water maze) in aging mice with sustained mitochondrial damage compared to their developing counterparts (20). Importantly, the current findings suggest that when accounting for increasing levels of SOD in the system, decreases in mitochondrial SRC predicted weaker planning-related beta oscillations in M1 and faster reaction times on the task. In addition, increased levels of superoxide mediated the relationship between mitochondrial function and movement-related beta power during the execution phase in the contralateral M1. Finally, decreases in SRC were predictive of faster reaction times and in turn, faster movement durations across our sample. While the integrity of the mitochondria and its associated functional properties (e.g., oxygen consumption, ATP production, and Ca^2+^ sequestering) have been implicated in neurophysiological changes in health and disease (12, 14, 17), our study suggests that features of the redox environment (i.e., superoxide and SOD) are critical modulators of the brain-behavior dynamics serving human motor control. Furthermore, our findings demonstrate that these superoxide-sensitive redox parameters are not merely byproducts of mitochondrial metabolism but exhibit dissociable mechanisms of action on planning and execution-related neural oscillatory activity serving motor function in M1, which had yet to be reported in the context of human neurophysiology.

Mechanistically, our mitochondrial, redox-regulated beta oscillatory responses could be attributable, at least in part, to changes in GABAergic inhibitory interneurons. Briefly, there is substantial evidence implicating GABAergic-mediated inhibitory drive in the modulation of pyramidal cells, giving rise to specific oscillatory responses including the generation of higher-frequency beta and gamma oscillations (26, 27, 48–50), especially within the motor and visual networks (31–33). In regard to the redox environment, the generation of higher-frequency oscillators requires the most energy expenditure in the brain (23, 24), and pharmacologically disrupting superoxide and H_2_O_2_-sensitive features of this environment has direct consequences on the integrity of inhibitory interneuronal pools, including substantial losses in absolute number of interneurons detected as well as decreased parvalbumin expression in animal models (22). Thus, we propose that the mitochondrial, redox-regulated mechanisms observed in the current study could be acting through modulation of GABAergic inhibitory interneuronal pools to modulate the strength of population-level neural oscillations in the beta range. Future work in this area will be critical to fully unravel the nature of bioenergetic–oscillatory coupling in the sensorimotor cortices and to possibly extend these observations to gamma–frequency oscillations, which our cued motor task did not reliably elicit.

Our study directly links quantitative measures of mitochondrial respiration and the redox environment to the neural oscillatory responses underlying motor function in healthy adults. Importantly, our results provide evidence that the beta oscillations serving motor planning and execution are modulated by the redox environment and sensitive to superoxide redox parameters as opposed to H_2_O_2_-pertinent mechanisms. In fact, we observed both direct and mediating effects of superoxide-sensitive mitochondrial redox features that accounted for 34.9% of the variance in behavioral performance on the motor sequence task, while we observed no predictive paths on brain or behavioral function in humans when considering H_2_O_2_-sensitive redox parameters. Future work could pursue direct intracellular quantification of H_2_O_2_ to clarify the involvement of H_2_O_2_ in human behavior, albeit our results currently suggest a lack of involvement of H_2_O_2_-sensitive scavengers in human motor control. Further, while our results implicated mitochondrial energetic reserves and associated superoxide and SOD involvement in modulating brain and behavior in the current sample, future work would greatly benefit from the quantification of ROS and antioxidants directly within the mitochondria (e.g., SOD2: mitochondrial isoform of SOD) to corroborate our findings on mitochondrial-specific redox environments in human neurophysiology. Concomitant with these experimental prospects to motivate future work, human studies should also carefully consider the various participant-related factors that may induce individual variability in systemic redox biology. These factors vary widely and may include environmental exposures to pollutants or substances (e.g., recreational drug use) (51, 52), daily regimens including diet and exercise (e.g., obesity) (53), and clinical status such as the level of immune compromise (54–56), neurodegeneration (14–16), or even participant age (13, 21, 57). Thus, it will be of utmost importance for continued work in the field to carefully control for factors that have demonstrated notable changes in these redox parameters. Additionally, future work should examine other oscillatory signatures pertinent to motor control. For example, in addition to peri-movement beta decreases in the motor system, there are robust increases in beta activity after movement (i.e., the postmovement beta rebound). While this increase in beta power may simply reflect the motor cortex returning to idling levels, this response has also been linked to motoric inhibition and/or somatosensory reafference to the motor cortex (58–60). Other spectral changes involved in motor control include low (i.e., theta) and high (i.e., gamma) frequency oscillations that occur during movement onset and importantly may serve as temporal coordinators (61, 62) or execution signals for volitional movement (33, 63, 64), respectively. Although recent work has also demonstrated that such gamma oscillations are modulated by higher-order processes such as response interference and attentional reorienting (65–69). While the current study focused on the impact of the mitochondrial redox environment on peri-movement beta responses due to their established relationship with behavior (1–4, 8, 9, 11, 43, 60, 69), we did probe the other oscillatory responses in our supplementary analyses and found that the effects were generally specific to beta oscillations during motor planning and execution. Relatedly, the current study also examined motor-related responses in the emerging beta-burst framework. Surprisingly, we observed no relationship between the mitochondrial redox environment and the two most-common beta-burst event parameters (i.e., event count and peak event power), again suggesting at least some specificity to the temporally sustained beta oscillations serving motor planning and execution. Future work using different motor tasks and samples could help establish this specificity to peri-movement beta oscillations or extend the effects to other oscillations and/or bursting activity.

En masse, our study demonstrated a strong link between mitochondrial function, the redox environment, and brain and behavioral function in healthy adults. Specifically, decreases in mitochondrial energetic capacities were predictive of weaker peri-movement beta responses in the contralateral primary motor cortices and, further, faster reaction times and shorter movement durations through discrete elevations in superoxide and its antioxidant scavenger, SOD. To close, our study suggests that molecular precursors to brain-behavior dynamics (i.e., blood-based markers of mitochondrial redox pathways) as evaluated in the current study may provide critical insights to behavior and brain function in humans and, further, may serve as effective targets in the future to ameliorate age- and disease-related declines in cognitive function in adults.

## Materials and Methods

### Participant Demographics.

A total of 40 adults (M_age_ = 45.1 y old, range: 20 to 66 y old; 17 females, 37 right-handed) were enrolled in the current study. Exclusion criteria included any medical illness affecting central nervous system function, any psychiatric or neurological disorder, history of head trauma, current pregnancy, and current substance use. The University of Nebraska Medical Center Institutional Review Board approved the study, and all participants provided written informed consent.

### Experimental Paradigm.

Participants were seated in a nonmagnetic chair with their head positioned within the MEG helmet-shaped sensor array. Participants were initially asked to fixate on a crosshair for 3,750 ms (± 350 ms), followed by a presentation of three numbers for 500 ms, each corresponding to a finger on the hand (Fig. 1). Upon the presentation of a visual cue (i.e., numbers turning blue), participants were asked to tap the fingers corresponding to the numbers sequentially and given 2,250 ms to complete the movement. A total of 160 trials were completed, making the overall recording time ∼17 min.

### MEG Data Acquisition and Coregistration with Structural MRI.

All recordings were performed in a one-layer, magnetically shielded room with active shielding engaged for environmental noise compensation. With an acquisition bandwidth of 0.1 to 330 Hz, neuromagnetic responses were sampled continuously at 1 kHz using a MEGIN/Elekta MEG system (MEGIN, Helsinki, Finland) with 306 magnetic sensors, including 204 planar gradiometers and 102 magnetometers. Throughout data acquisition, participants were monitored using a real-time audio–video feed from inside the magnetically shielded room. MEG data from each participant were individually corrected for head motion and subjected to noise reduction using the signal-space separation method with a temporal extension (70). Each participant’s MEG data were coregistered with their structural T1-weighted MRI data prior to imaging analyses using Brain Electrical Source Analysis (BESA) MRI (Version 2.0). Structural MRI data were aligned parallel to the anterior and posterior commissures and transformed into standardized space. After beamformer analysis (see *MEG Source Imaging* below), each subject’s functional images were transformed into standardized space using the transform that was previously applied to the structural MRI volume and spatially resampled.

### MEG Preprocessing and Sensor-Level Statistics.

Cardiac and ocular artifacts were removed from the data using signal-space projection, and the projection operator was accounted for during source reconstruction (71). Epochs of 6,150-ms duration were defined (i.e., −2,050 to 4,100 ms) with 0 ms defined as the onset of the first movement in the sequence and the baseline being the −2,050- to −1,550-ms window. Initially, we rejected trials based on having a reaction time longer than 1,250 ms or taking more than 3,000 ms to complete the entire motor sequence, which would disrupt the baseline period of the subsequent trial. Next, epochs containing artifacts were rejected based on a fixed threshold method, supplemented with visual inspection. On average, 123 trials per participant were used for further analysis.

Artifact-free epochs were transformed into the time–frequency domain using complex demodulation (72), and the resulting spectral power estimations per sensor were averaged over trials to generate time–frequency plots of mean spectral density. The sensor-level data per time–frequency bin were normalized using the mean power within that frequency bin during the −2,050- to −1,550-ms baseline period. The specific time–frequency windows used for imaging were determined through a two-stage, data-driven approach involving statistical analysis of the sensor-level spectrograms across all participants and trials. First, paired-sample *t* tests against baseline were conducted on each data point, with the output spectrogram of t-values initially thresholded at *P* < 0.05 to define time–frequency bins containing potentially significant oscillatory deviations. To reduce the risk of false-positive results due to multiple comparisons, the time–frequency bins that survived that initial threshold were temporally and/or spectrally clustered with neighboring bins that were also significant, and a cluster value was derived by summing all of the t-values of all data points in the cluster. Nonparametric permutation testing (10,000 permutations) was then used to derive a distribution of cluster values, and the significance level of the observed clusters were tested directly using this distribution. Based on this analysis, the time–frequency periods that contained significant oscillatory events across all participants were subjected to beamforming analyses. Of note, in the case of the temporally sustained peri-movement beta oscillations, we focused on a window surrounding the peak of the response (i.e., greatest amplitude change from baseline) in order to optimize the signal to noise ratio and selected, equally sized 500-ms temporal windows for the motor planning and execution periods to facilitate the interpretation of findings. Note that the significant time–frequency extent of the peri-movement beta response extended beyond this 1,000-ms period. Further details of our MEG data processing pipeline can be found in recent manuscripts (43, 68, 69, 73).

### MEG Source Imaging.

Cortical oscillatory responses were imaged through the dynamic imaging of coherent sources beamformer (74), which uses the cross-spectral density matrices to calculate source power for the entire brain volume. These images are typically referred to as pseudot maps, with units (pseudot) that reflect noise-normalized power differences (i.e., active versus passive) per voxel. Following convention, we computed noise-normalized source power per voxel in each participant using baseline periods of equal duration and bandwidth (75). MEG preprocessing and imaging used the BESA software version 7.0.

Normalized source power was computed over the entire brain volume per participant at 4.0 × 4.0 × 4.0-mm resolution for the time–frequency periods identified through the sensor-level analyses. Prior to statistical analysis, each participant’s MEG data, which were coregistered to native space structural MRI prior to beamforming, were transformed into standardized space using the transform previously applied to the structural MRI volume and spatially resampled. The resulting three-dimensional maps of brain activity were averaged across all participants to assess the neuroanatomical basis of each oscillatory response identified through the sensor-level analysis. Source power was then extracted from peak voxels per time bin (i.e., planning and execution phases) per participant and underwent statistical modeling. For the analysis of beta bursting activity, we extracted the time series of this same peak voxel, and this was subjected to a standard beta-burst analysis pipeline that is described in *SI Appendix*.

### Isolation of Peripheral Blood Mononuclear Cells and Respiration Analysis.

Whole blood was collected into ethylenediaminetetraacetic acid (EDTA) tubes by venous puncture for all participants. Buffy coats were submitted to a Ficoll-Paque Plus (GE Healthcare) gradient centrifugation for isolation of the mononuclear fraction. Peripheral blood mononuclear cells (PBMCs) were cryopreserved in Fetal Bovine Serum with 10% dimethyl sulfoxide (DMSO). Cells were thawed within 6 wk of isolation and underwent assessment using the Seahorse XF96 Analyzer (Seahorse Bioscience) to quantify oxygen consumption rate (OCR) using the mitochondrial stress test assay. Specifically, PBMCs were plated at 500,000 cells/well, and three OCR measurements were taken sequentially on five to six technical replicate wells prior to and upon serial injection of 3.5 μM oligomycin (Sigma-Aldrich; complex V inhibitor), 1 μM fluoro-carbonyl cyanide phenylhydrazone (FCCP; Sigma-Aldrich; mitochondrial oxidative phosphorylation uncoupler), and 14 μM rotenone + 14 μM antimycin A (Sigma-Aldrich; complex I and III inhibitors, respectively) to evaluate measures of mitochondrial respiration including basal respiration, ATP-linked respiration, proton leak, maximal respiration, SRC and nonmitochondrial respiration. All bioenergetic data were normalized to protein in the well for subsequent analyses. For data calculation, the Seahorse Wave software (version 2.2.0) was used.

### Quantification of the Redox Environment.

Cellular levels of superoxide were assessed using EPR spectroscopy of whole blood incubated with a superoxide-sensitive spin probe (1-hydroxy-3-methoxycarbonyl1-2,2,5,5-tetramethylpyrrolidine: CMH) for 1 h under physiologic conditions (37 °C), as previously described (76). Of note, the CMH spin probe was chosen for the current protocol for several reasons including its cell permeability as well as its temporal characteristics, which are superior to spin trapping methods. EPR spin traps stabilize the free radical that they trap; however, they are still relatively short lived in biological samples (i.e., whole blood) as they are quickly reduced to their nonradical form. In contrast, the CMH spin probe reacts directly with superoxide yielding the CM radical (CM^•^), which is stable at biological pH and temperature for at least 4 h. This allows us to process the whole-blood sample and obtain EPR spectra to examine levels of superoxide in the sample. In addition, the cell permeability of CMH allows for the detection of superoxide within subcellular compartments (e.g., mitochondria), making this a promising method for future interrogations in this domain (i.e., mitochondrial redox biology). Immediately after sample collection, 200 μM of CMH was reconstituted into EPR buffer (Krebs–Hepes Buffer) supplemented with metal chelators (5 μM sodium diethyldithiocarbamate trihydrate [DETC] and 25 μM deferoxamine [DF]) and incubated with 200 μL of whole blood. Importantly, the concentrations of DETC and DF (5 μM and 25 μM, respectively) were carefully chosen to improve the efficacy of these metal chelators to minimize the influence of metals reacting with more unstable free radicals in our sample. EPR measurements were performed with a Bruker eScan EPR spectrometer (Bruker BioSpin GmbH, Rheinstetten/Karlsruhe, Germany), with the following parameters: field-sweep width, 100.0 G; center field, 3482 G; microwave frequency, 9.75 kHz; microwave power, 1.10 mW; modulation amplitude, 5.94 G; conversion time, 10.24 ms; and time constant, 40.96 ms. The resulting EPR spectra amplitude is expressed as arbitrary units that are directly proportional to the amount of total cellular superoxide in the sample.

Antioxidant activity levels were quantified in erythrocytes for key enzymatic and nonenzymatic contributors to the mitochondrial redox environment, including SOD, catalase, and glutathione. Specifically, we used the SOD Assay Kit-WST (DOJINDO, Inc.) to measure total SOD activity, the OxiSelect Catalase Activity Assay Kit (Cell Biolabs, Inc.) for catalase, and the GSSG/GSH Quantification kit (DOJINDO, Inc.) for oxidized (GSSG), reduced (GSH), and total glutathione levels (tGSH; i.e., GSSG + GSH) according to the manufacturers’ guidelines.

### Statistical Analysis.

To evaluate the predictive capacity of the mitochondrial redox environment on movement-related neural oscillations serving motor function and behavior, we used structural equation modeling following standard data trimming procedures. Specifically, bioenergetic, redox, neural, and behavioral data underwent standard data evaluation protocols such that measures exceeding 2.5 SDs above or below the sample’s mean were excluded from subsequent analyses. Next, we evaluated assumptions of normality by assessing the skewness/kurtosis of our metrics and performed log transformations for those exhibiting skewness/kurtosis values ± 1.0 (i.e., indicative of nonnormal distributions) where appropriate. Our primary hypotheses were that movement-related beta oscillatory activity during planning and execution phases of movement would predict task performance and that these dynamics would be modulated by the mitochondrial redox environment. In regard to the brain and behavior, we predicted that beta activity prior to movement (i.e., planning) would predict reaction time and beta decreases in the contralateral M1 during motor execution. These variables would then predict the time to complete the entire motor sequence (i.e., movement duration). In addition, we conducted a multiple-mediation model whereby measures of mitochondrial function (i.e., SRC) predicted movement-related oscillations and subsequent behavioral performance through changes in the redox environment (i.e., superoxide, SOD, catalase, and GSSG/tGSH ratio) for superoxide-sensitive and H_2_O_2_-sensitive paths, separately. Importantly, we examined the 95% CIs of bias-corrected bootstrapped confidence intervals based on 1,000 bootstrapped samples (42). This method provides a robust estimate of mediation effects that are asymmetrical (41), as traditional tests of indirect effects (e.g., Sobel test) often violate the assumption of normality. All analyses were conducted with full information maximum likelihood estimation for missing data using MPlus (version 8.1). For more details regarding our structural equation modeling and statistical procedures, refer to *SI Appendix*.

Finally, to facilitate comparison with recent work investigating motor cortical beta activity within the transient, bursting event framework, we conducted supplementary analyses evaluating the predictive capacity of superoxide- and H_2_O_2_-sensitive mitochondrial redox environments on event characteristics during the baseline period (i.e., −2,050 to −1,550 ms) and behavior on the task. Using code adapted from Shin et al., we tested a model whereby measures of the mitochondrial redox environment predicted beta-burst event count and peak event power (35), which would subsequently predict reaction time and movement duration on the motor sequence task. For a full description of the methods and a summary of the regression results, refer to *SI Appendix*.

## Data Availability

MEG recordings from each participant (.fif), structural MRI data (.nii), and mitochondrial redox measure (.csv) data have been deposited in the institutional website of Boys Town National Research Hospital (https://cdn.boystown.org/media/misc/Spooner_PNAS_2021_Data.zip).
